# The development of microfabricated solenoids with magnetic cores for micromagnetic neural stimulation

**DOI:** 10.1038/s41378-021-00320-8

**Published:** 2021-11-12

**Authors:** Adam Khalifa, Mohsen Zaeimbashi, Tony X. Zhou, Seyed Mahdi Abrishami, Neville Sun, Seunghyun Park, Tamara Šumarac, Jason Qu, Inbar Zohar, Amir Yacoby, Sydney Cash, Nian X. Sun

**Affiliations:** 1 Department of Neurology, Massachusetts General Hospital, Harvard Medical School, Boston, MA USA; 2 Department of Electrical and Computer Engineering, Northeastern University, Boston, MA USA; 3 Research Laboratory of Electronics, Massachusetts Institute of Technology, Cambridge, MA USA; 4 Department of Physics, Harvard University, Cambridge, MA USA; 5 Department of Chemical and Biological Physics, Weizmann Institute of Science, Rehovot, Israel

**Keywords:** Electronic properties and materials, Electronic devices

## Abstract

Electrical stimulation via invasive microelectrodes is commonly used to treat a wide range of neurological and psychiatric conditions. Despite its remarkable success, the stimulation performance is not sustainable since the electrodes become encapsulated by gliosis due to foreign body reactions. Magnetic stimulation overcomes these limitations by eliminating the need for a metal-electrode contact. Here, we demonstrate a novel microfabricated solenoid inductor (80 µm × 40 µm) with a magnetic core that can activate neuronal tissue. The characterization and proof-of-concept of the device raise the possibility that micromagnetic stimulation solenoids that are small enough to be implanted within the brain may prove to be an effective alternative to existing electrode-based stimulation devices for chronic neural interfacing applications.

## Introduction

Implanting electrodes to stimulate excitable tissue has been a viable therapeutic strategy for treating human disorders for many decades. For instance, deep brain stimulation (DBS) has been successful in the treatment of movement disorders such as Parkinson’s disease. Moreover, clinical trials are currently underway to examine the efficacy of DBS for the treatment of additional neurological and psychiatric diseases, including epilepsy, mood disorders, and obsessive-compulsive disorder^1–4^.

Despite the successes of direct electrical stimulation, chronically implanted electrodes face decreased performance over time^5–7^. Therapeutic effects can be altered by inflammatory and immune reactions of the tissue in response to direct contact with the stimulating electrode, since glial scarring around the stimulating electrode will eventually increase stimulation thresholds. Increasing the stimulation intensity to circumvent this issue causes larger reduction and oxidation reactions at the electrode-tissue interface, which leads to damage to both the electrode and the surrounding tissue^8,9^.

Implantable micromagnetic stimulation (µMS) has recently been suggested as a new form of brain stimulation. Recent works^10–12^ have shown that submm- and mm-scale coils are capable of converting an applied electric current into magnetic flux, which then induces an electric field gradient strong enough to move the ions and propel them to induce (or inhibit) responses in neurons.

Unlike electrodes, the conductive materials used during µMS are not in contact with neural tissue. Moreover, unlike transcranial magnetic stimulation (TMS), µMS uses small implantable coils, thus achieving a much higher spatial and temporal resolution. Therefore, a contact-less neural stimulation tool such as micromagnetic coils could potentially improve the long-term functionality of neural interfacing devices.

In 2012, Bonmassar et al. showed that a commercially available inductor (500 µm in diameter and 1 mm long) can activate neuronal tissue^13^. Soon after, multiple types of miniaturized inductors and coils were developed^11,12,14,15^, some of which were made to be used invasively to increase the spatial resolution^10^. The overarching goal of this research is to rely on microfabrication technologies to fabricate efficient µMS probes that could overcome some of the limitations of conventional electrode-based stimulation devices. In this research, we present microfabricated solenoid inductors with magnetic cores that significantly amplify the generated magnetic flux compared to air-core microcoils. Although the presented probes are more difficult to microfabricate than those presented in past works, the larger magnetic flux density that they generate allows the following: (1) further reduction in the size of µMS probes, which will increase spatial resolution, (2) a decrease in the amount of current delivered to the probe, which will decrease the heat generated by the inductor, or (3) activation of a larger number of neurons around the probe. In this manuscript, we first briefly discuss the important parameters that should be considered when designing a micromagnetic stimulation coil. We then describe the steps toward the construction of the microsolenoid inductor with a magnetic core (~80 × 40 µm^2^) and discuss its advantages compared to air-core microcoils. We created a computational finite element method (FEM) model that allowed us to study the magnetic/electric fields arising from µMS. We then measured the magnetic flux density using a state-of-the-art microscale magnetometer tool based on a nitrogen-vacancy (NV) center in diamond. To explore whether such small solenoids could indeed evoke neural activity, we conducted an ex vivo calcium fluorescence imaging (GCaMP6s) experiment. In short, for the first time, we describe the microfabrication of these probes, their magnetic flux measurements, and their proof-of-concept in brain slices. Although further validation using animal models is required, this research focuses on the microfabrication and characterization of novel ultrasmall solenoids optimized for neural stimulation.

## Materials and methods

### Microsolenoid microfabrication procedure

**Fig. 1:**
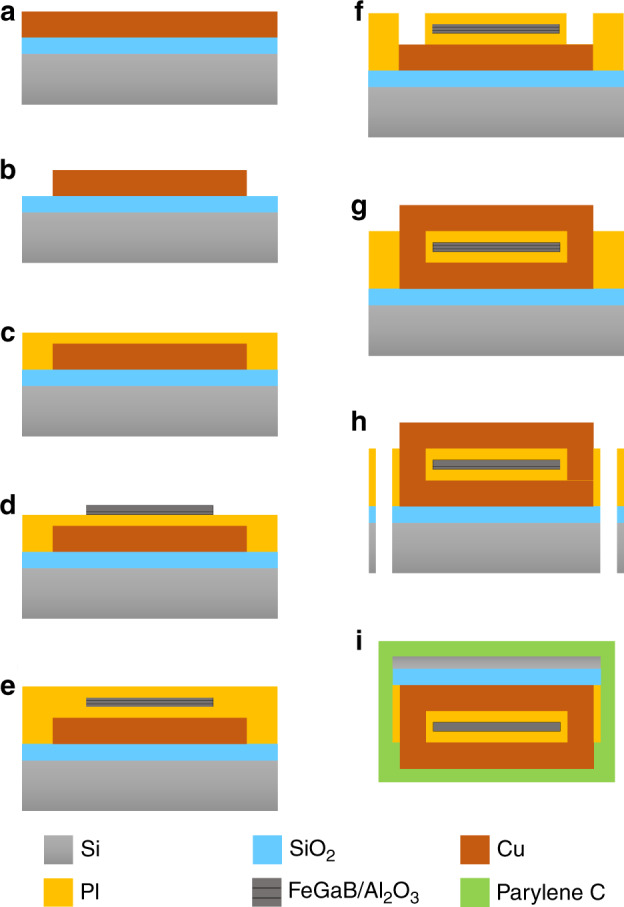
Fabrication process flow of the microsolenoid with a magnetic core. **a** The seed layer was deposited and patterned. **b** Cu was deposited on SiO_2_ by electroplating. **c** PI was deposited and cured. **d** FeGaB/Al_2_O_3_/FeGaB was deposited and patterned using lift-off. **e** PI was deposited again and cured. **f** Cr was deposited to pattern the PI, which was etched using ICP. **g** Cu was deposited again by electroplating. **h** Si and SiO_2_ were etched using DRIE. **i** The probe was flipped and the Si thinned. The entire probe was then coated in Parylene C

The microfabrication of the solenoid inductors is described in this section (Fig. 1). Microsolenoid coils were fabricated on a 4-inch 500 µm thick Si wafer with 500 nm SiO_2_. To form the conductive wires, a 100 nm seed layer was deposited by physical vapor deposition (PVD) and patterned using photolithography. Then, 3 µm thick copper (Cu) was deposited by electroplating, which was done by applying a current of 100 mA for 1 hour. In this work, we chose Cu as the inductor winding material since it has high electrical and thermal conductivity and is compatible with standard microfabrication processes. Although Cu is toxic to tissue, the material is fully encapsulated and therefore isolated from tissue. A 4 µm thick polyimide (PI) PI2611 was then spin-coated and baked at 350 °C for 45 min for curing. In addition to isolating the Cu coil from the conductive magnetic core, the PI layer was also used to fill the gap between the copper windings to make a flat surface for the following magnetic film deposition. The multiferroic layer was then deposited using PVD by a magnetron sputtering method. The magnetic core is a sandwich structure of FeGaB (100 nm)/Al_2_O_3_ (5 nm)/FeGaB (100 nm) repeated 15 times, resulting in a total thickness of 3 µm. Laminated magnetic cores help minimize eddy currents, and FeGaB was chosen for its very high relative permeability. The magnetic core was then patterned using a lift-off process. Another spin-coating step was used for the top PI layer, but this time, the curing temperature was set to 250 °C to protect the magnetic films. Chromium (Cr), which acted as a hard mask, was deposited directly onto polyimide and patterned by photolithography and a lift-off process. Inductively coupled plasma reactive ion etching (ICP-RIE) was then used to form vias in the PI layer, which exposed the bottom Cu coil. The same electroplating step was then used for the top Cu coil. As a result, windings could be formed as the top and bottom Cu were connected through Cu filling in the vias. Deep reactive ion etching (DRIE) was used to etch the Si and SiO_2,_ which released the microprobes from the 4-inch substrate. After removing the Cr layer, the probes were flipped to thin the Si substrate using DRIE. The probes were then cleaned with acetone and isopropanol and carefully handled using a vacuum pickup pen for placement and gluing onto the printed circuit board (PCB).

**Fig. 2:**
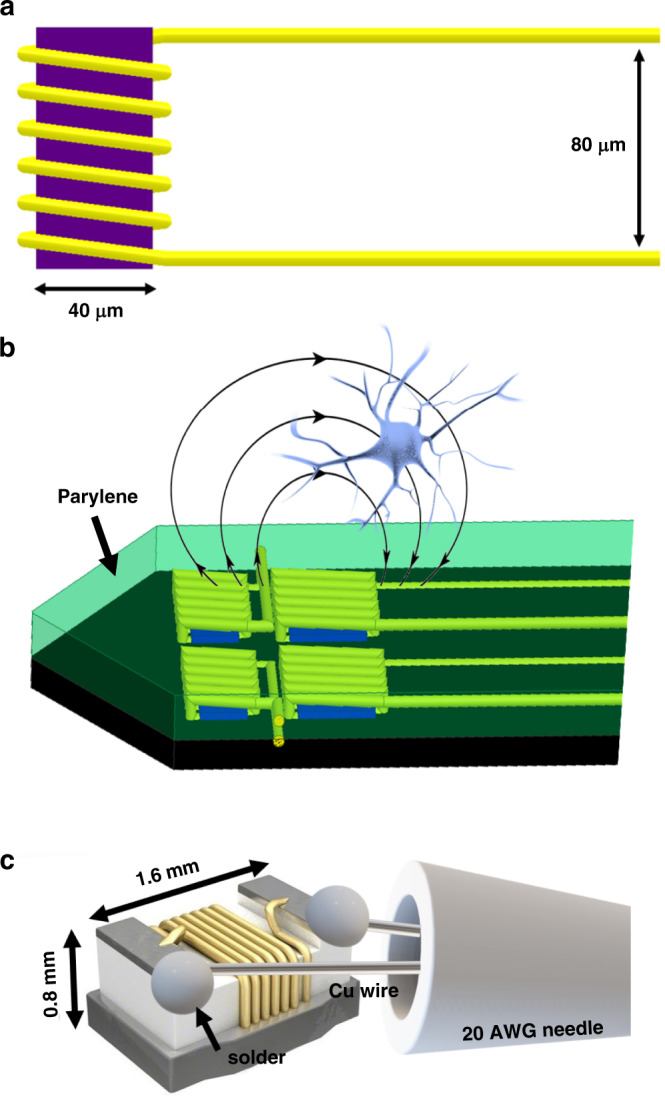
3D drawings of the microsolenoid and macrosolenoid. **a** The microsolenoid and its dimensions. **b** A silicon probe with four microsolenoids at the tip encapsulated with Parylene C. **c** The macrosolenoid probe and its dimensions (acrylic coating is not shown)

Next, using a wedge-wedge wire bonding machine (25 µm Al wire), the copper pads on the microprobes were connected to the pads on the PCB to enable the delivery of current to the microcoils. Finally, the entire assembly was coated with ~10 µm of Parylene C for encapsulation. To enable penetration into the brain, the probes measured 100–200 µm in width and 30 µm in thickness and were fabricated on rigid silicon substrates (such as neuropixels and other CMOS compatible probes). Some of the silicon substrates had a length that measured up to 6 mm, allowing the solenoids to reach any brain region of a rodent model. The designed probes had multiple inductors with different specifications along the length of the probe, such as different sizes and orientations. The inductors used in this research measured 80 µm × 40 µm (Fig. 2).

### Macrosolenoid probe assembly

To test the robustness of our fluorescence calcium imaging setup and to verify that the acute slice was healthy enough to respond to magnetic stimulation, we decided to use a previously validated technology. The goal was to reconstruct a larger magnetic probe that has been shown to induce neural activity in past work. In this work, we define macrosolenoid probes as mm- to submm-scale solenoids, such as those utilized in past work^11–13^. Macrosolenoids do not offer the same stimulation focality as microsolenoids and are too invasive to serve as intracortical probes.

The assembly of the macrosolenoid probe, which is composed of a surface-mount device (SMD) inductor, was inspired by the work of Bonmassar et al.^13^. We used a submillimeter commercially available inductor (LQW18ANR10G0ZD, Murata, Japan) with an inductance value of 100 nH and a resistance value of 0.68 Ω. Two 0.12 mm diameter Cu wires were inserted into the shaft of a 20 AWG needle and the barrel of a syringe. The plunger of the syringe was replaced with a BNC connector that was connected using a glue gun and connected to the wires. After soldering the SMD inductor to the Cu wires, we coated the macrosolenoid and the wires with an acrylic conformal coating (MG chemicals).

### Electromagnetic simulations

We used the magnetic and electric field (mef) module in COMSOL Multiphysics to analyze magnetic and electric field behavior in the low-frequency regime. This module uses FEM to solve the partial differential equations. The formulas governing the magnetic and electric fields generated by coils are Maxwell’s equations.

It is difficult to estimate the electric field gradient threshold for neuromodulation, as it is linked to multiple parameters^16,17^.

Therefore, our target when designing the was to confirm through FEM simulations that the generated electric field gradients of the proposed solenoid are much higher than those of a conventional flat coil (which was shown to be able to stimulate neurons in prior work^10^). Several parameters define the inductance of a coil. The most important ones are (a) the number of turns, (b) the material of the core, (c) the cross-sectional area of the coil, and (d) the length of the coil. While designing a microscale size inductor, there are some limitations in terms of the number of turns and cross-sectional area of the coil. A successfully designed microsolenoid is one that generates a strong E-field gradient in a certain direction. The microsolenoid contained a 3 µm thick core made of FeGaB, a composite magnetic material with a high relative permeability. The relative permittivity, relative permeability, and electrical conductivity of FeGaB used in simulations were 1, 990, and 1.2E6 S/m, respectively. The solenoid inductors have 6 turns. A half-cycle alternative current (100 mA, 5 kHz) was applied to the microsolenoids.

**Fig. 3:**
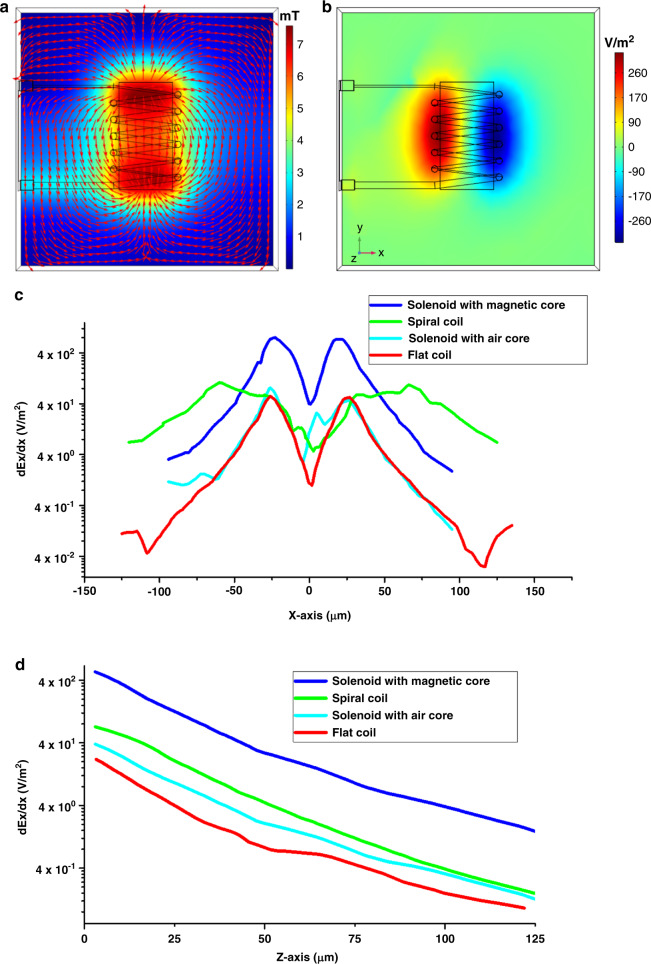
FEM simulations of the designed microsolenoid with a magnetic core show its electric field gradient distribution and how it compares to other types of coils/solenoids. **a** Magnetic flux density distribution of the microsolenoid on a *z* = 10 µm plane. **b** Electric field gradient (dEx/dx) distribution of the microsolenoid on the *z* = 10 µm plane. **c** dEx/dx for different types of inductors along the *x*-axis on the *z* = 2 µm plane. **d** dEx/dx for different types of inductors along the z-axis

Fig. 3 shows some of the FEM simulation results of a 40 × 80 µm inductor. At a z distance of 10 µm, the magnetic flux density reaches 8 mT, whereas the induced electric field gradient peak is ~270 V/m^2^. In previous works^18^, we compared the performance of microsolenoid inductors with a magnetic core with flat-coil (single-turn), spiral coil, and microsolenoids inductors with an air core (Fig. S1). Fig. 3c, d shows the Ex component for different microcoil/microsolenoids along the *x*-axis and *z*-axis. The FEM simulations show that the dEx/dx component of the proposed microsolenoid is >15 times larger than that of the three other types of inductors.

### Experimental setup for magnetic flux density measurements

**Fig. 4:**
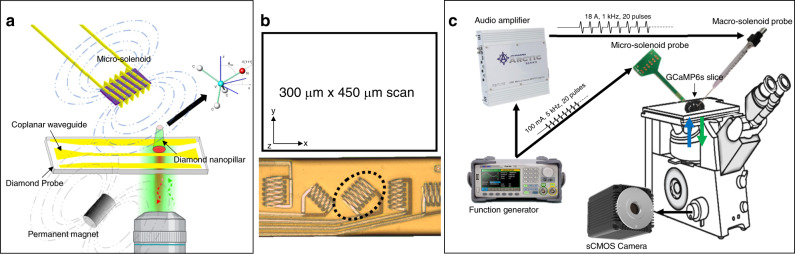
Drawing of the experimental setups. **a** The setup used for measuring the magnetic flux density emitted by the novel microsolenoid using a custom-built system based on an NV diamond sensor. **b** The scan window is shown used in setup (**a**). **c** The setup used proof-of-concept of µMS using the micro/macrosolenoids in acute brain slices

To verify the accuracy and reliability of the simulation results, we measured the magnetic flux distribution generated by a microsolenoid. Unfortunately, this is not a trivial task, as commercially available magnetic field sensors (such as Gauss meters) do not have the spatial resolution to visualize the magnetic field generated by ultrasmall solenoids. Therefore, microscale imaging was performed using a quantum sensing technique based on a single NV center in diamond. It consists of nitrogen substitution of one carbon atom and a neighboring carbon vacancy with an electron spin of 1. Via control and readout of its quantum state, the NV detects local magnetic flux with atomic resolution. This magnetometer is remarkable in terms of its high sensitivity and its ability to be functional in a wide range of frequencies. The experimental setup is shown in Fig. 4a. A diamond chip was prepared via previous methods^19^ and underwent processes^20^ to define a nanopillar to contain a single NV at the apex. We then fabricated a coplanar waveguide (CPW)^21^ onto the surface of a diamond chip to deliver a microwave signal for NV spin state control. An inverted confocal setup for efficiently collecting NV photoluminescence was designed and built for this research. The NV center was optically excited with a 532-nm laser, which allows pulsed excitation and initiation of NV. The excitation light was exerted onto the NV center through an Olympus 0.9 NA ×100 objective and the emitted photons were also collected through the same objective. The emitted light was collected after passing through a 552-nm edge dichroic filter (Semrock LM01-552-25), followed by another 570-nm long-lass filter. A cylindrical NdFeB permanent magnet (K&J D48N52) was used to apply a static external magnetic field. The microsolenoid probe was fixed onto a motorized translation stage (Thorlabs KDC101, MTS25-Z8). The microwave signal to control the NV spin state was applied through the CPW by a microwave generator (Rhode Schwartz SMB100A). All timing control, including the current flowing through the microsolinoid, was performed by a Tektronix Arbitrary Waveform Generator 5014C. The interval of the pulsed AC current was 1 µs.

Our NV measurement scheme was based on AC magnetometry with a large bandwidth generally used from 1 kHz to 10 MHz and a $10nT/\sqrt {Hz}$ sensitivity, as shown in recent experiments^22,23^. This magnetometry can also be applied to image signal above GHz signal^24^. Magnetic imaging was implemented by measuring the magnetic flux density projected in the direction of the NV axis (we denote this field component as B_NV_) at each pixel and moving the coil on the *x–y* plane with a fixed *z* height to construct the image. Note that the NV axis was in the direction of the straight line connecting the nitrogen atom and vacancy inside the diamond lattice (crystallographic <111> direction, see Fig. 4a, right inset). The microsolenoid and the diamond were aligned so that the axis of the NV center was in the *y–z* plane of the lab frame, as indicated in Fig. 4b.

Measurements were taken at multiple distances between the microsolenoid and the diamond (*z* height), including 40 µm, 60 µm, and 140 µm. To avoid the risk of touching the diamond nanopillar, the probe was placed on its side. As shown in Fig. 4b, the scan windows measured 300 µm × 450 µm. The magnetic flux density around the microsolenoid was measured while applying 25 mA of current. We did not use larger currents to avoid risking burning of the solenoid. Note that it was possible to use larger currents during ex vivo experiments since the solenoid was inserted in aCSF, which limited the temperature rise of the probe.

### Experimental setup for the proof-of-concept in acute brain slices

Calcium imaging setups were used to validate the technology for two main reasons: (1) they allow us to explore the spatial extent of µMS, and (2) they are immune to electromagnetic interference (caused by the large amount of power transmitted by the inductor), unlike electrophysiology setups that rely on microelectrodes for neural recordings. All research protocols were approved and monitored by the Massachusetts General Hospital (MGH) Institutional Animal Care and Use Committee (IACUC). Ex vivo calcium fluorescence imaging experiments were conducted using brain slices extracted from 15- to 30-day old transgenic mice (Thy1-GCaMP6s, Jackson Laboratory, ME, USA). The mice were deeply anesthetized with isoflurane and decapitated. Three hundred-micrometer-thick slices were prepared using a vibratome and incubated in aCSF solution at 34 °C. After a one-hour recovery period, the slices were transferred to the recording chamber, which was continuously perfused with oxygenated aCSF solution. Images were captured using an sCMOS camera (Dhyana 400BSI, Tucsen Photonics, Fujian, China) mounted on an inverted wide-field fluorescence microscope (Ts2R-FL, Nikon Instruments Inc, NY). A ×10 objective was used for the microsolenoid, whereas a ×20 objective was used for the macrosolenoid. Images were analyzed using image analysis software (ImageJ, National Institute of Health). Transients were measured as the ratio of the peak amplitude of the transient (ΔF) to the averaged baseline value (F) measured before the stimulus.

The proof-of-concept experimental setup is shown in Fig. 4c. We used a micromanipulator to carefully insert the microsolenoid into the brain slice to a depth of ~290 µm and at an angle of ~55°. This meant that the solenoid was ~170 µm deep into the brain slice. We then applied µMS stimuli that were generated using a function generator (SDG2042X, Siglent, Ohio). The stimulus waveform delivered to the solenoid was a train of 20 pulses delivered at a rate of 1 Hz. Each pulse consisted of a single full-period 5 kHz sinusoidal waveform. The current amplitude delivered to the inductor was calculated to be approximately ±100 mA. The DC resistance of the powered microsolenoid was measured during the experiment to confirm the working conditions of the solenoid. A resistance larger than 20 Ω indicates that the path from the PCB bond pad to the solenoid was damaged. Furthermore, before and after each experiment, we tested the probes for current leakages by measuring the impedance (at 1 kHz) between one of the two terminals of the probe and a large electrode. Both the probe and the electrode were submerged into a saline bath. If the measured impedance was found to be below 10 MΩ, then the probe would be deemed defective and would not be tested in acute slices. In addition, we measured the temperature of the probe using the stimulation parameters and waveform described above. The temperature profile was captured using a high-resolution infrared thermal camera (VarioCAM HD head 900, Infratec). Since infrared wavelengths do not transmit well through aCSF and tissue, the temperature of a probe floating in air was recorded.

The microsolenoid probe was also held by a micromanipulator. However, since the probe was too large to be inserted into the slice, the SMD inductor was positioned ~10 µm above the tissue slice, and its long axis was oriented parallel to the slice surface. The microsolenoid probe was driven by a 1000 W audio amplifier (PB717X, Pyramid Inc., NY), which was connected to a function generator (SDG2042X, Siglent, Ohio). The signal provided to the macrosolenoid was similar to that provided to the microsolenoid with the exception of much higher current peaks (~ ±18 A) and a slightly lower frequency (1 kHz). The current was estimated by measuring the voltage across the inductor, which was monitored using an oscilloscope.

## Results and discussion

### Fabricated prototype

**Fig. 5:**
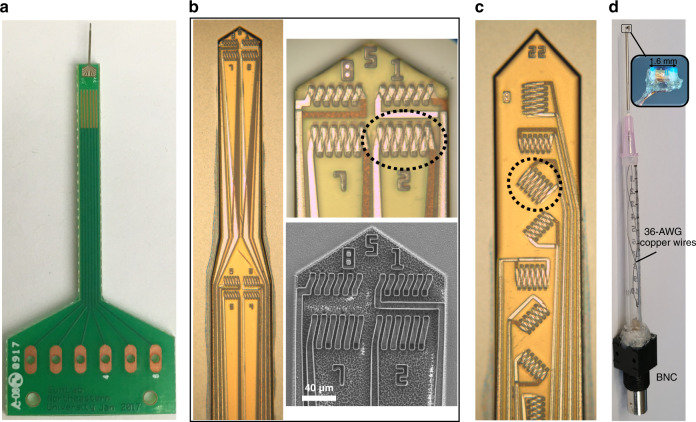
Fabricated probes for µMS. **a** Silicon probe mounted on a PCB board. **b** Optical microscope images of the probes used during the ex vivo brain slice experiment. Close-up views (optical and SEM) of the tip are also shown to better visualize the solenoids. **c** Optical microscope image of the fabricated probe used during magnetic flux measurement. **d** Picture of the macrosolenoid probe used during the ex vivo brain slice experiment with a close-up view of the macrosolenoid. The microsolenoids used during the experiments are encircled in black

Fig. 5a, b shows a picture of the assembled probe and SEM and optical microscope images of the microfabricated silicon probe used in this research. We fabricated 23 different probes, each numbered near the tip of the probe. Some probes, such as probe #15, have inductors measuring 40 × 40 µm in size to provide higher spatial resolution (Fig. S2). Probe #5 was used during the ex vivo brain slice experiment. It has similar inductors across the length of the probe, which could be useful for in vivo stimulation of neuronal regions located at different depths in the brain. This is another advantage the presented prototype has over previously validated microcoil probes, which only have the option to stimulate at one site^10^. We would like to add that we have not encountered any interference between the solenoids, as their coupling is weak and the applied frequency signal is not large enough to leak between traces. Probe #22 (Fig. 5c) has 8 inductors with different orientations. This probe was designed to investigate the impact of different magnetic flux directions on the surrounding neurons. Although the silicon probe used during the ex vivo brain slice experiment (Fig. 5b) is different from that used during the magnetic flux measurement (Fig. 5c), the same type of microsolenoid was used, which measured 40 × 80 µm with an impedance of 11 Ω (1 kHz). Fig. 5d shows the assembled macrosolenoid probe used during the ex vivo brain slice experiment. The resistance of the entire probe is measured to be 1.3 Ω.

### Magnetic flux density measurements

**Fig. 6:**
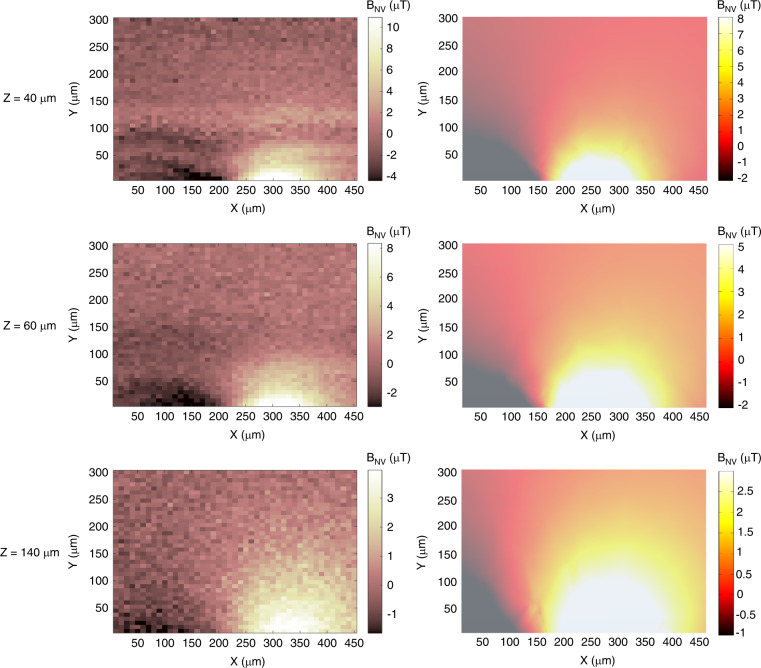
The magnetic flux density below a microsolenoid. Microsolenoid magnetic flux density distribution obtained from (left) measured results and (right) FEM simulations for 3 different distances (40, 60, and 140 µm) between the solenoid and the diamond sensor

The measurement results of the magnetic flux density distribution of the microsolenoid are shown in Fig. 6. The measurement and simulation results match the flux distribution and magnetic flux density values. The measured magnetic flux density is ~20% higher than the simulated density. The small discrepancy is most likely due to the inaccuracy of the *z* values since estimating the distance between the solenoid and the diamond nanopillar at the micron scale is challenging. It can be concluded that our FEM-simulated electric field gradient distribution previously shown in Fig. 3b is also accurate, as it is directly linked to the magnetic flux density.

### Neural stimulation using the macrosolenoid and microfabricated solenoid

**Fig. 7:**
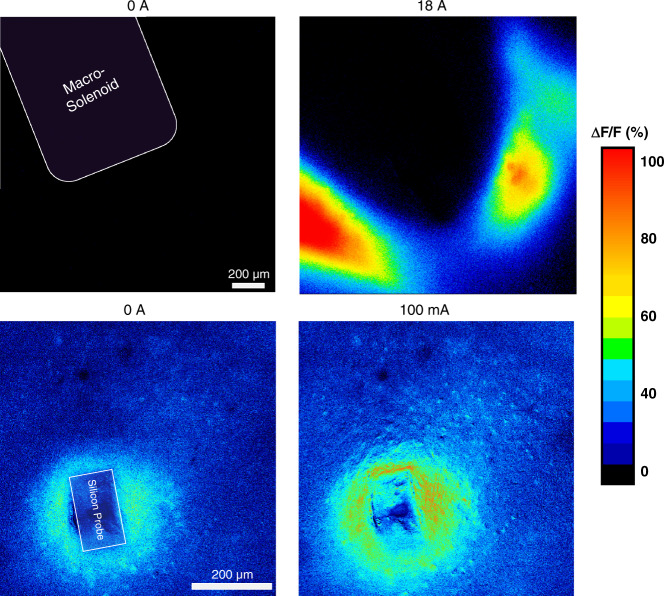
Proof-of-concept of magnetic stimulation. Epifluorescence micrograph of a brain slice from Thy1-GCaMP6s transgenic mice showing the change in fluorescence in response to µMS when using a (top) macrosolenoid and a (bottom) microsolenoid

Before evaluating the responses of cortical neurons to µMS using the proposed microsolenoid, we first measured the responses via a macrosolenoid. As shown in Fig. 7(top), there was a strong increase in fluorescence (ΔF/F = 100%). The strongest response seems to appear near the edges of the inductor. As expected, the spatial extent (<200 µm) and the response are smaller (ΔF/F < 90%) with the microsolenoid as the spatial resolution is much higher; thus, the population of neurons that is activated is only a few microns away from the solenoid (Fig. 7(bottom)). Most of the neurons neighboring the solenoid cannot be seen using a simple epifluorescence microscope. Bringing the microsolenoid closer to the slide would not have been possible without breaking the fragile silicon probe. It should be noted that fluorescence images with the microsolenoid show a relatively high level of baseline noise, which explains the light blue color background in 7(bottom). This is caused by the continuous minuscule vibrations of the silicon caused by perfusion of aCSF through the chamber. Slice anchors would mitigate this issue; however, we decided not to use one to avoid coupling between the inductor and the metal loop, which was part of the slice anchor. We also monitored the temperature of the microsolenoid probe when floating in air and measured a maximum increase of 0.7 °C (Fig. S3), which is well below the threshold for thermal activation of neurons^25^ and tissue damage^26^. Since the tissue and aCSF cooling rate is much higher than that of air, we expect a much smaller temperature rise when the probe is implanted. Although we have demonstrated experimentally that µMS can modulate neuronal activities, further studies are needed to understand the relationship between the parameters of magnetic stimulation and neuronal activation as well as the influence of solenoid spatial orientation on the neuronal response. We did not attempt to explore this further, as this manuscript focuses on the microfabrication and characterization of ultrasmall inductors with magnetic cores. The next iteration of this work will involve chronic experiments in awake behaving animals to show that microsolenoids are more stable and safer than intracortical stimulating electrodes, which would bring this technology one step closer to neurological and neuropsychiatric clinical applications.

## Conclusion

As demonstrated by recent works, µMS has several advantages over electrode-based stimulation. Improvements in nanofabrication technology have allowed us to create ultrasmall solenoids with magnetic cores that can generate larger magnetic fields while being completely encapsulated in a biocompatible coating. This research presents, for the first time, the nanofabrication steps to construct a working prototype, the measured magnetic flux induced by the solenoid, and the magnetic stimulation proof-of-concept in acute slices. Our novel microfabricated solenoid successfully activated neural tissue and therefore shows potential as a viable alternative to current neural interfacing tools for basic neuroscience and clinical applications, although further investigation is required.

## Supplementary information

Supplemental material

## References

[1] Widge AS (2017). Treating refractory mental illness with closed-loop brain stimulation: progress towards a patient-specific transdiagnostic approach. Exp. Neurol..

[2] Widge AS, Dougherty DD (2015). Deep brain stimulation for treatment-refractory mood and obsessive-compulsive disorders. Curr. Behav. Neurosci. Rep..

[3] Lozano AM, Lipsman N (2013). Probing and regulating dysfunctional circuits using deep brain stimulation. Neuron.

[4] Andrade DM (2006). Long-term follow-up of patients with thalamic deep brain stimulation for epilepsy. Neurology.

[5] Liu X (1999). Stability of the interface between neural tissue and chronically implanted intracortical microelectrodes. IEEE Trans. Rehabilitation Eng..

[6] Biran R, Martin DC, Tresco PA (2005). Neuronal cell loss accompanies the brain tissue response to chronically implanted silicon microelectrode arrays. Exp. Neurol..

[7] Polikov VS, Tresco PA, Reichert WM (2005). Response of brain tissue to chronically implanted neural electrodes. J. Neurosci. Methods.

[8] Merrill DR, Bikson M, Jefferys JGR (2005). Electrical stimulation of excitable tissue: design of efficacious and safe protocols. J. Neurosci. Methods.

[9] Khalifa A, Gao Z, Bermak A, Wang YI, Chan LLH (2015). A novel method for the fabrication of a high-density carbon nanotube microelectrode array. Sens. Bio-Sens. Res..

[10] Lee SW, Fallegger F, Casse BDF, Fried SI (2016). Implantable microcoils for intracortical magnetic stimulation. Sci. Adv..

[11] Minusa S, Osanai H, Tateno T (2017). Micromagnetic stimulation of the mouse auditory cortex in vivo using an implantable solenoid system. IEEE Trans. Biomed. Eng..

[12] Golestanirad L (2018). Solenoidal micromagnetic stimulation enables activation of axons with specific orientation. Front. Physiol..

[13] Bonmassar G (2012). Microscopic magnetic stimulation of neural tissue. Nat. Commun..

[14] Park H-J (2013). Activation of the central nervous system induced by micro-magnetic stimulation. Nat. Commun..

[15] Lee, S. W. Selective activation of cortical columns using multichannel magnetic stimulation with a bent flat microwire array. *IEEE Trans. Biomed. Eng.* **68**, 2164–2175 (2021). 10.1109/TBME.2020.3033491 PMC8262115 33095707

[16] Maccabee PJ, Amassian VE, Eberle LP, Cracco RQ (1993). Magnetic coil stimulation of straight and bent amphibian and mammalian peripheral nerve in vitro: locus of excitation. J. Physiol..

[17] Åström M, Diczfalusy E, Martens H, Wårdell K (2014). Relationship between neural activation and electric field distribution during deep brain stimulation. IEEE Trans. Biomed. Eng..

[18] Zaeimbashi, M. et al. Micro-solenoid inductors with magnetic core for neural stimulation. In *2018 40th Annual International Conference of the IEEE Engineering in Medicine and Biology Society (EMBC)*, 2230–2233. (IEEE, 2018). 10.1109/EMBC.2018.8512729 30440849

[19] Dovzhenko Y (2018). Magnetostatic twists in room-temperature skyrmions explored by nitrogen-vacancy center spin texture reconstruction. Nat. Commun..

[20] Xie L, Zhou TX, Stöhr RJ, Yacoby A (2018). Crystallographic orientation dependent reactive ion etching in single crystal diamond. Adv. Mater..

[21] Zhou TX, Stöhr RJ, Yacoby A (2017). Scanning diamond nv center probes compatible with conventional afm technology. Appl. Phys. Lett..

[22] Ku MJH (2020). Imaging viscous flow of the dirac fluid in graphene. Nature.

[23] Vool, U. et al. Imaging phonon-mediated hydrodynamic flow in WTe2. *Nat. Phys.*10.1038/s41567-021-01341-w (2021).

[24] Zhou TX (2021). A magnon scattering platform. Proc. Natl Acad. Sci. USA.

[25] Chen R, Romero G, Christiansen MG, Mohr A, Anikeeva P (2015). Wireless magnetothermal deep brain stimulation. Science.

[26] Yarmolenko PS (2011). Thresholds for thermal damage to normal tissues: an update. Int. J. Hyperth..

