# Supplementary material for: The development of microfabricated solenoids with magnetic cores for micromagnetic neural stimulation
Source: Microsyst Nanoeng. 2021 Nov 12;7:91. doi: 10.1038/s41378-021-00320-8 (PMC8589949; doi:10.1038/s41378-021-00320-8)
Supplement: Supplementary file 1 — Supplemental material [file 41378_2021_320_MOESM1_ESM.docx]

**Supplementary Materials:**

The Development of Microfabricated Solenoids with Magnetic Cores for Micromagnetic Neural Stimulation

Adam Khalifa1*, Mohsen Zaeimbashi1,2*, Tony X. Zhou3,4, Seyed Mahdi Abrishami2, Neville Sun2, Seunghyun Park4, Tamara Šumarac4, Jason Qu4, Inbar Zohar5, Amir Yacoby4, Sydney Cash1, and Nian X. Sun2

**
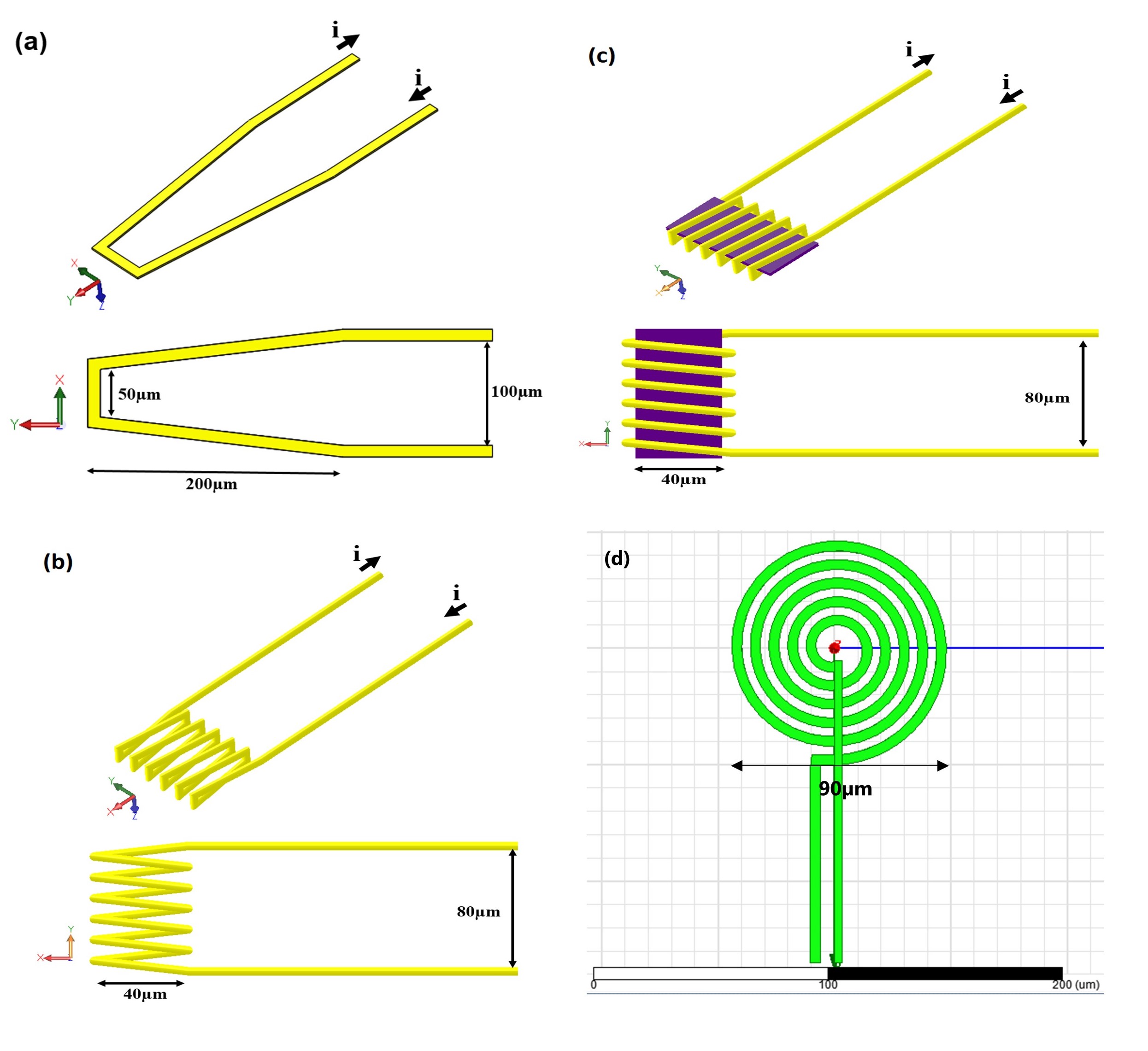
**

S1: Geometry of simulated coils/solenoids. (a) Flat coil which is a single wire structure with two sharp corners at the tip. (b) Solenoid inductor with air-core. (c) Solenoid inductor with FeGaB core. (d) Spiral coil.


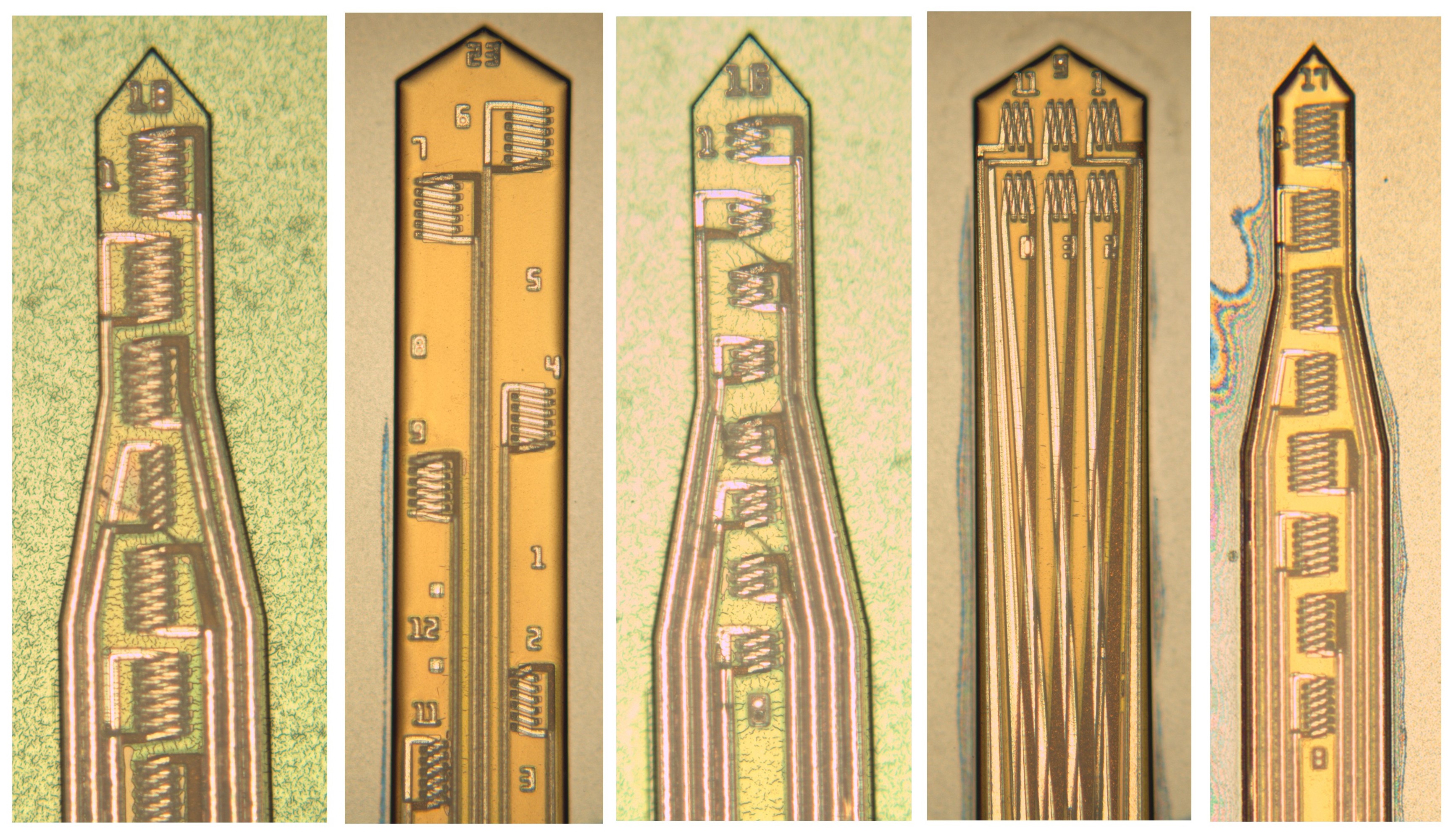


S2: Optical microscope images of different microfabricated probes with different inductors sizes, configuration, and orientation to tackle multiple neural interfacing applications and needs.


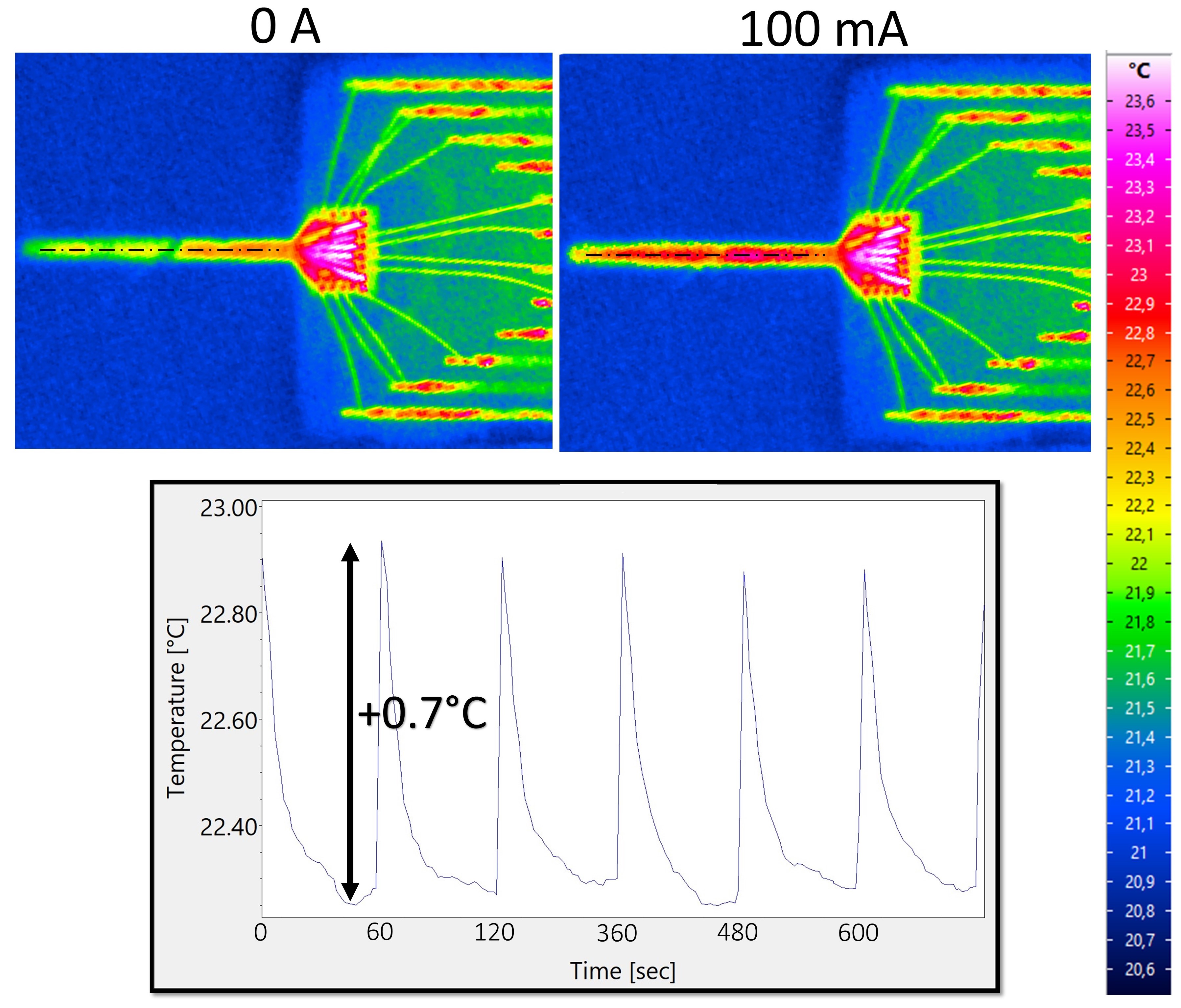


S3: Thermal temperature measurements using an infrared thermal camera of the micro-solenoid probe during magnetic stimulation (train of 20 pulses delivered at a rate of 1 Hz). (Top) Thermal pictures before and during stimulation. (Bottom) Average temperature measurement over time of the probe. The measured area is displayed as a dotted black line on the thermal pictures.
